# Genomic characterization of ER-positive primary tumors and corresponding relapses identifies potentially targetable alterations

**DOI:** 10.1038/s41523-026-01041-9

**Published:** 2026-08-30

**Authors:** Caroline Schagerholm Stanev, Stephanie Robertson, Hosein Toosi, Emmanouil G. Sifakis, Jens Lagergren, Johan Hartman

**Affiliations:** 1 Department of Oncology-Pathology, Karolinska Institutet, Stockholm, Sweden; 2 Division of Computational Science and Technology, KTH Royal Institute of Technology and Science for Life Laboratory, Stockholm, Sweden; 3 Department of Clinical Pathology and Cancer Diagnostics, Karolinska University Hospital, Stockholm, Sweden

**Keywords:** Biomarkers, Cancer, Genetics, Oncology

## Abstract

The majority of breast cancer patients have tumors expressing estrogen receptor α (ER) and receive endocrine therapy. However, around one-third relapse in their disease, predominantly with retained ER expression. Molecular alterations are proposed to be contributors to the resistance mechanisms. Patients with ER-positive, human epidermal growth factor receptor 2 (HER2)-negative primary breast cancer with an ER-positive relapse < 5 years of ongoing endocrine therapy were retrospectively assessed. Extracted DNA was analyzed through panel sequencing, and RNA by microarray, from patients’ primary (*n* = 58), and paired relapse tumors (*n* = 54), and tumor-free lymph nodes (DNA germline controls, *n* = 62). Several single-nucleotide variations and copy number variations showed nominal exploratory associations with worse overall survival. Copy number correlations with intrinsic subtypes and individual gene expression supported the findings. These results identify hypothesis-generating genomic and transcriptomic features, including potentially targetable alterations, in a clinically defined cohort of endocrine-resistant breast cancer patients.

## Introduction

Each year, over 2.3 million persons receive a breast cancer diagnosis globally^1^. Out of these, around three-fourths have tumors expressing estrogen receptor α (ER) and are consequently offered endocrine therapy. Resistance to treatment remains a major issue, as up to 50% of patients experience questionable benefit. Additionally, in the majority of patients with a breast cancer relapse, ER expression is retained in the tumor^2–8^. ER is, however, the only clinically utilized marker for endocrine therapy, and novel tools are needed^3,7–11^.

At the genomic level, several studies have investigated the mutational landscape of primary and metastatic breast cancer. However, material on paired primary and relapse tumor samples is rare^12–14^. Existing studies have shown mutational concordance rates of 75% or higher between primary and metastatic tumor samples in various cancers, as well as in breast cancer^15–17^. Albeit this number shows variations depending on different alterations, and absolute concordance has been revealed to be as low as 18%^12,18,19^. Issues arising from tumor heterogeneity and clonal evolution of the recurrent tumor complicate the evaluation of concordance. Thus, the prevalence of enriched mutations justifies recommendations for diagnostic molecular testing of the recurrent tumor in the clinical setting for treatment guidance^20,21^.

*ESR1* mutations have been found in up to 40% of metastatic breast cancer tumors^22,23^. Further acquisition of driver mutations in the advanced setting has been revealed, such as *TP53* and members of Janus kinase-signal transducer and activator of transcription (JAK-STAT) signaling, as well as phosphatidylinositol-4,5-bisphosphate 3-kinase catalytic subunit alpha (*PIK3CA*), GATA binding protein 3 (*GATA3*), and mitogen-activated protein kinase (MAPK)-pathway mutations^14,19,24–28^. Moreover, evaluations of paired ER-positive metastatic post-treatment samples from endocrine-resistant breast cancer patients have identified acquired mutations in the fibroblast growth factor (FGF) pathway, which is also associated with resistance to cyclin-dependent kinase 4 and 6 (CDK4/6) inhibitors^29^. Further, the AURORA study, investigating patients with paired samples of primary and metastatic breast cancer, showed significantly higher median tumor mutational burden (TMB) in metastatic samples^13^. A high TMB was moreover associated with a shorter time to relapse, and ESMO Scale for Clinical Actionability of molecular Targets (ESCAT)^30,31^ tier I/II mutations were prevalent in half of the patients^13^. However, questions on the analysis methodology and standardization of TMB cut-offs are debated, and a consensus is needed for the clinical use of TMB^32–35^.

Furthermore, copy number variations (CNVs) have been revealed as potential genetic drivers of disease^12^. Metastatic CNV signatures are shown to mirror primary tumors, though with increased alteration burden and more intermixed profiles^36,37^. In addition, the CNV burden has been associated with cancer prognosis^38^. Research has, however, also demonstrated that aberrations in the metastatic setting may not be actual drivers of tumorigenesis, but are instead associated with drug resistance^36,37,39^.

At the transcriptomic level, breast cancer tumors can be divided into intrinsic subtypes based on their gene expression patterns. The PAM50 signature involves 50 genes that distinguish breast cancer into subtypes with varying prognosis (Luminal A, Luminal B, HER2-enriched, Basal-like, and Normal-like)^40–42^. Tumors of the Luminal B subtype are associated with a worse prognosis than Luminal A and are suggested to be less estrogen-dependent and linked with endocrine resistance^43–45^. Mutations in *PIK3CA*, *GATA3*, and *MAP3K1* have, instead, been associated with tumors of the Luminal A subtype^46^.

Genomic and transcriptomic advances have thus revealed several potential mechanisms for endocrine resistance and identified plausible diagnostic tools. Nonetheless, difficulties persist in study methodologies, including the scarcity of paired samples, issues of confounders, and generalizability that is dependent on inclusion and exclusion criteria, as well as assessment cut-offs. Compared with previous large genomic studies, this study focused on a strictly defined cohort of patients with ER-positive human epidermal growth factor receptor 2 (HER2)-negative primary breast cancer tumors with paired ER-positive relapse tumors, during ongoing endocrine therapy and without anti-HER2 treatment. Furthermore, the study involved paired data on the DNA and RNA levels, allowing specific investigation of the resistance evolution under endocrine pressure. By examining the patients' clinicopathological, gene expression, and genomic profiles, the aim was to contribute to insights into proposed genomic and transcriptomic mechanisms of endocrine resistance and disease progression in breast cancer.

## Results

### Clinicopathological data

The clinicopathological characteristics are summarized in Table 1, together with the TMB, number of CNVs, and the intrinsic subtypes of the patients’ primary and relapse tumors. Further, patient and treatment information are summarized in Table 2.

**Table 1:** Clinicopathological characteristics, genomic burden, and intrinsic subtypes of the Endoresist cohort, divided into patients’ primary and relapse tumors

	Primary tumors *N* = 62^a^	Relapse tumors *N* = 62^a^
*Histological subtype*		
Ductal	43 (69.35%)	30 (48.39%)
Lobular	14 (22.58%)	11 (17.74%)
Other	5 (8.06%)	2 (3.23%)
N/A	0 (0.00%)	19 (30.65%)
*Histologic grade*		
NHG1	9 (14.52%)	5 (8.06%)
NHG2	28 (45.16%)	17 (27.42%)
NHG3	24 (38.71%)	14 (22.58%)
N/A	1 (1.61%)	26 (41.94%)
*Lymph node status*		
Negative	35 (56.45%)	20 (32.26%)
Positive	27 (43.55%)	11 (17.74%)
N/A	0 (0.00%)	31 (50.00%)
*ER*		
Median, % (range)	90 (5–100)	90 (20–100)
*PR*		
Median, % (range)	40 (0–100)	5 (0–100)
*PR status*		
< 10%	14 (22.58%)	27 (43.55%)
≥ 10%	35 (56.45%)	23 (37.10%)
N/A	13 (20.97%)	12 (19.35%)
*Ki67*		
Median, % (range)	20 (1–95)	20 (0–100)
*HER2* *status*^*b*^		
HER2-null	37 (59.68%)	28 (45.16%)
HER2-low	22 (35.48%)	28 (45.16%)
HER2-amplified	0 (0.00%)	1 (1.61%)
N/A	3 (4.84%)	5 (8.06%)
*TILs*		
Median, % (range)	5 (1–40)	10 (1–40)
*TMB*		
Median, mutations/Mb (range)	4.58 (0–20.83)	4.37 (0.83–37.5)
*CNV burden*		
Median, number per sample (range)	63.5 (0–272)	58 (0–326)
*PAM50 intrinsic subtype*		
Luminal A	31 (50.00%)	28 (45.16%)
Luminal B	22 (35.48%)	18 (29.03%)
HER2-enriched	2 (3.23%)	4 (6.45%)
Basal	2 (3.23%)	3 (4.84%)
Normal	1 (1.61%)	1 (1.61%)
N/A	4 (6.45%)	8 (12.90%)

*NHG* Nottingham Histologic Grade, *ER* estrogen receptor, *PR* progesterone receptor, *HER2* human epidermal growth factor receptor 2, *TILs* tumor-infiltrating lymphocytes, *TMB* tumor mutational burden, *Mb* megabase, *N/A* data not available.

^a^All samples, even if lacking DNA or RNA data.

^b^HER2-null  HER2 IHC score 0, HER2-low HER2 IHC score ≥ 1 and negative HER2 in situ hybridization (ISH), HER2-amplified  amplified by ISH.

**Table 2:** Patient and treatment information of the patients in the Endoresist cohort

	Patients *N* = 62^a^
*Age at primary diagnosis*	
Median, years (range)	61 (30-88)
*Surgical procedure primary tumor*	
Mastectomy	30 (48.39%)
Sector excision	31 (50.00%)
N/A	1 (1.61%)
*Adjuvant endocrine therapy*	
Tamoxifen	33 (53.23%)
Aromatase inhibitor	24 (38.71%)
Other	5 (8.06%)
*Adjuvant chemotherapy*	
Received	32 (51.61%)
Not received	30 (48.39%)
*Adjuvant radiotherapy*	
Received	40 (64.52%)
Not received	22 (35.48%)
*Relapse location*	
Ipsilateral	22 (35.48%)
Contralateral no DCIS	5 (8.06%)
Contralateral to DCIS	14 (22.58%)
Distant metastasis	21 (33.87%)
*Distant metastasis location (N = 21)*	
Colorectal	1 (4.75%)^b^
Contralateral axilla	1 (4.75%)^b^
Liver	6 (28.57%)^b^
Lung	2 (9.52%)^b^
Skeleton	6 (28.57%)^b^
Skin	3 (14.29%)^b^
Ovary	2 (9.52%)^b^

*DCIS* ductal carcinoma in situ, *N/A* data not available.

^a^All samples, even if lacking DNA or RNA data.

^b^Percentages out of the patients with distant metastases (*N* = 21).

### Single-Nucleotide Variations

Investigating the overall mutational landscape of the 62 verified endocrine-resistant patients, a total of 1563 single-nucleotide variations (SNVs) were identified; 778 in the primary tumors and 785 in the relapse tumors. Of the mutations in the patients’ primary samples, 210 were found together with at least one other mutation in the same gene, while 568 were unique. In relapse tumors, 257 multiple and 528 unique mutations were found.

**Fig. 1:**
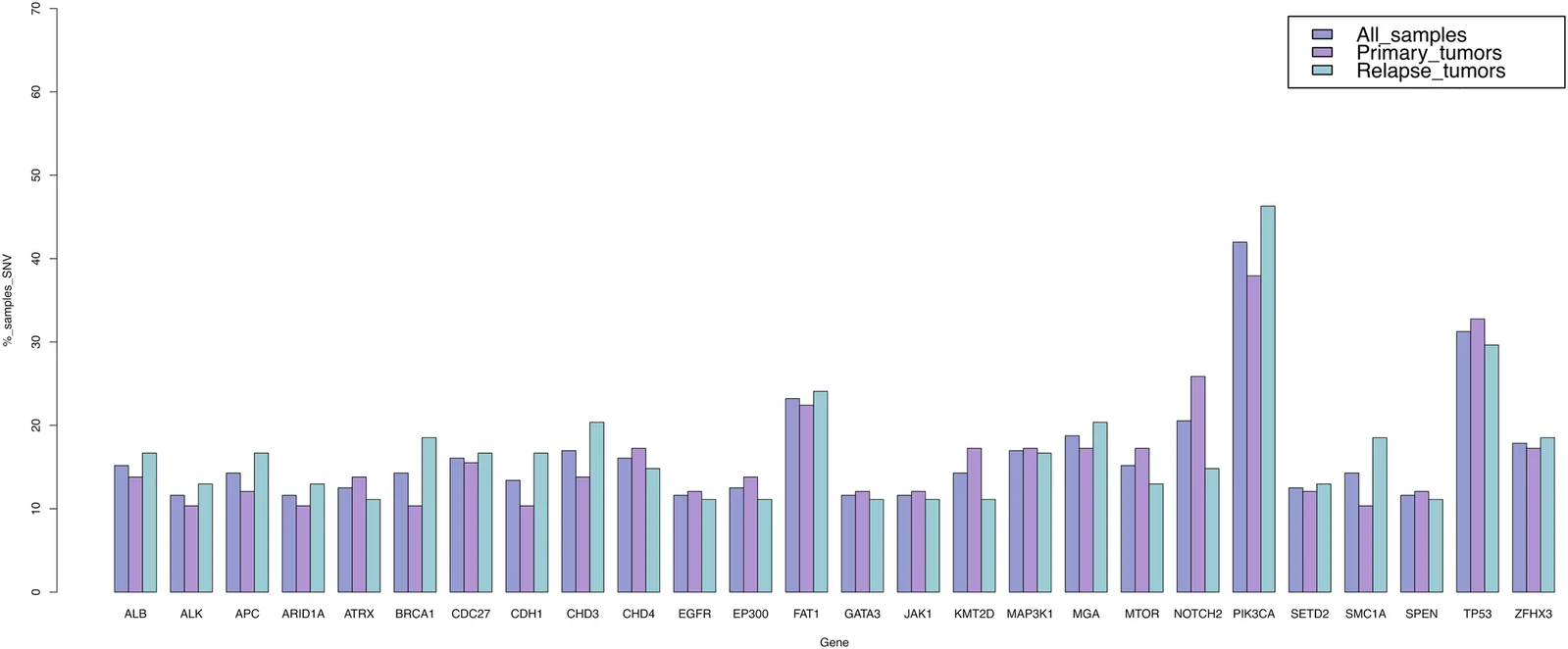
Single-nucleotide variations (SNVs) across selected genes in the Endoresist cohort. Bar plots of the SNV frequency for all samples (purple), primary tumors (plum), and relapse tumors (blue) per gene. Selected genes filtered by > 5 mutated samples of either the patients’ primary or relapse tumors.

The most commonly mutated gene was *PIK3CA*, where SNVs were found in 30/62 (48.39%) patients, followed by *TP53* in 22/62 (35.48%), *FAT1* in 20/62 (32.26%), *NOTCH2* in 20/62 (32.26%), and *MGA* and *ZFHX3* in 18/62 (29.03%). Divided on the patients’ tumor samples, the most commonly mutated genes in primary tumors were: *PIK3CA* in 22/58 (37.93%), *TP53* in 19/58 (32.76%), *NOTCH2* in 15/58 (25.86%), *FAT1* in 13/58 (22.41%), and *CHD4*, *KMT2D*, *MAP3K1*, *MGA*, *MTOR*, and *ZFHX3* in 10/58 (17.24%). In relapse tumors, the most commonly mutated genes were *PIK3CA* in 25/54 (46.30%), *TP53* in 16 (29.63%), *FAT1* in 13/54 (24.07%), *CHD3* in 11/54 (20.37%), and *MGA* in 11/54 (20.37%). The frequency of the most common SNVs, divided per the patients’ tumor samples, is visualized in Fig. 1. SNVs in *ESR1* were not found in the patients’ relapse tumors, and were present in two of the patients’ primary tumors.

### Copy number variations

Further, examining the CNVs, a total of 9026 were detected in all samples, of which 4964 (55.00%) were found in patients’ primary tumors and 4062 (45.00%) in relapse tumors. Out of the CNVs, 5696 (63.11%) were amplifications, and 3330 (36.89%) were deletions. Divided by the tumor group, 3083 (62.11%) were amplifications and 1881 (37.89%) deletions in patients’ primary tumors, and 2613 (64.33%) were amplifications and 1449 (35.67%) deletions in patients’ relapse tumors.

**Fig. 2:**
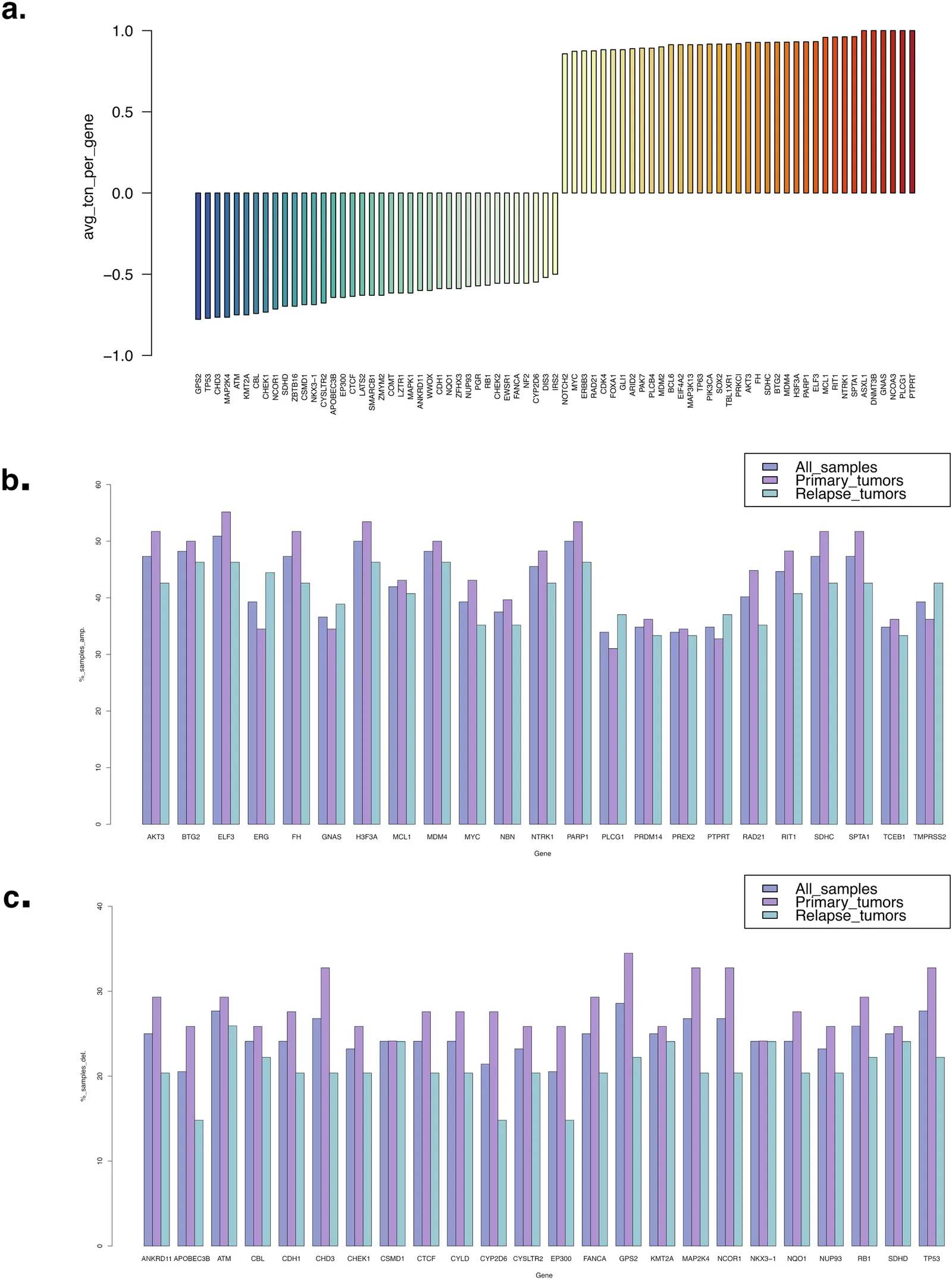
Bar plots illustrating the frequency of the copy numbers in the Endoresist cohort. The average total copy number (TCN) per gene found in the patients’ tumor samples, sorted by the lowest and highest (**a**). Bar plots of the frequency of amplifications (**b**) and deletions (**c**) in all samples (purple), primary tumors (plum), and relapse tumors (blue) of the patients, per gene. Selected genes filtered by an amplification in over one-third (> 33.33%) of patients’ samples or a deletion in over one-fifth (> 20%) of patients’ samples.

To evaluate the consequence and distribution of the CNVs, the total copy number (TCN)s was assessed. The genes with the highest and lowest average TCN across the samples, and a division of amplifications and deletions of CNVs, are visualized in Fig. 2. Of the genes, 23 demonstrated an amplification in over one-third (> 33.33%) of all tumor samples (Fig. 2b), and 24 demonstrated a deletion in over one-fifth (> 20%) of the patients’ samples (Fig. 2c).

### Mutual exclusivity analysis

The mutual exclusivity and co-occurrence analysis showed no significant comparisons in terms of *q*-value for the SNVs. Comparisons with *p*-value < 0.1 are available in Supplementary Table 1, presented together with BH-adjusted *q*-values.

### Tumor progression analysis

Out of the SNVs, 173 were shared between primary and relapse, 555 were gained in the relapse setting, 484 were lost from primary to relapse, and the remaining 178 were present in orphan samples. Out of the shared mutations, 106/173 (61.27%) had higher variant allele frequencies (VAFs) in the primary tumor, and 67/173 (38.73%) in the relapse tumor. When comparing the shared mutations VAFs by the Wilcoxon signed rank test, SNVs in eight genes (*CDC27*, *CDH1*, *FAT1*, *GATA3*, *HLA-B*, *PIK3CA*, *PTCH1*, and *TP53*) were present in at least three patients, in both primary and relapse tumors. Out of these, none demonstrated significant differences between the tumor settings at either *p*-values or BH-adjusted *q*-values.

Investigating the impact of CNVs between primary and relapse tumors of the patients, the Wilcoxon paired analysis of the TCN demonstrated 41 genes that were altered at a nominal *p*-value < 0.05 between the two tumor settings. Four genes (*AKT3*, *ELF3*, *FH*, and *SOX9*) showed a higher TCN in the primary tumors, and 37 genes (such as *CDH1*, *CHEK1*, *ESR1*, *MAP2K4*, *NCOR1*, and *TP53*) had higher TCN in the relapse tumors. After multiple testing correction, five genes (*CHD3*, *GPS2*, *MAP2K4*, *NCOR1*, and *TP53*) demonstrated BH-adjusted *q*-values < 0.1, all with higher TCN in the relapse tumors. The list of genes with *p*-value < 0.05, along with BH-adjusted *q*-values, are stated in Supplementary Table 2).

### Survival analysis

Evaluating the OS of patients in relation to the individual genes’ SNVs and CNVs through unadjusted and exploratory analyses, the presence of seven SNVs in the primary tumors and three in relapse tumors showed nominal associations with a worse overall survival (OS) for the patients. For CNVs, a worse OS was observed for amplifications in 62 genes in primary tumors and 13 in relapse tumors, and for deletions in 11 genes in primary and three in relapse tumors (Supplementary Table 3). When *p*-values were adjusted for using the Benjamini–Hochberg (BH) correction for multiple testing, 20 alterations remained with a BH-adjusted *q*-value < 0.1 (Supplementary Table 3). These involved amplifications in seven genes in patients’ primary tumors, including *ALK* and *MYCL*, deletions in three genes in primary tumors, including *NF1*, SNVs in *CHD3* in primary tumors, and amplifications in nine genes in relapse tumors, such as *IGF1R*, *MAP2K1*, and *RAD51*.

**Fig. 3:**
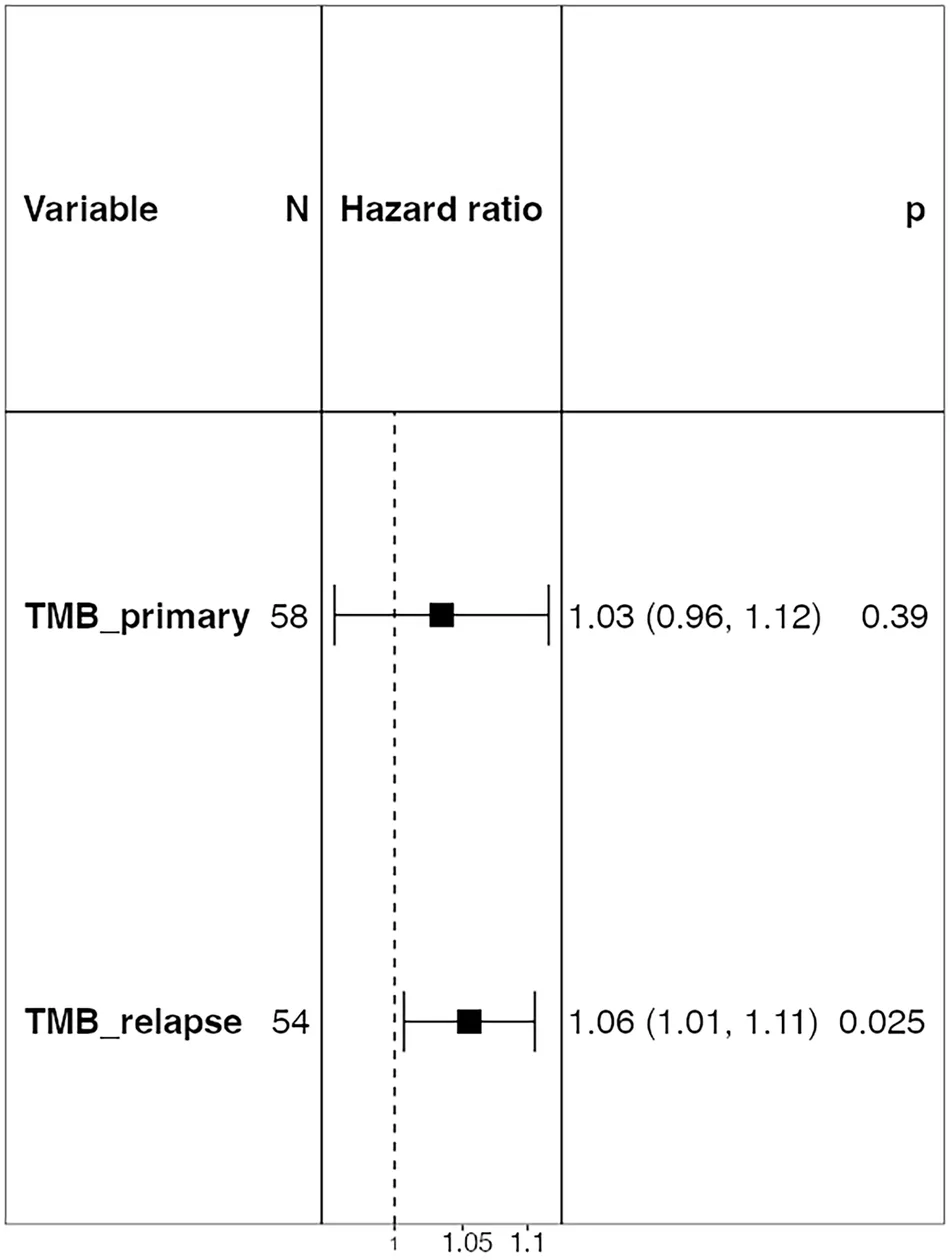
Forest plots depicting the association of tumor mutational burden with overall survival. Visual demonstration of the exploratory Cox proportional hazards univariate analyses of the tumor mutational burden (TMB) in patients’ primary and relapse tumors compared to overall survival.

The assessment of the continuous TMB values in patients’ relapse tumors showed an exploratory association with OS in the Cox proportional hazards univariate analyses (*p* = 0.025, Fig. 3). An association was not seen in the analysis of patients’ primary tumors (*p* = 0.39, Fig. 3). When investigated at the exploratory cut-off of 10, no association of TMB with OS was seen in either the patients primary or relapse tumors (log-rank *p* = 0.94 and log-rank *p* = 0.07, respectively).

**Fig. 4:**
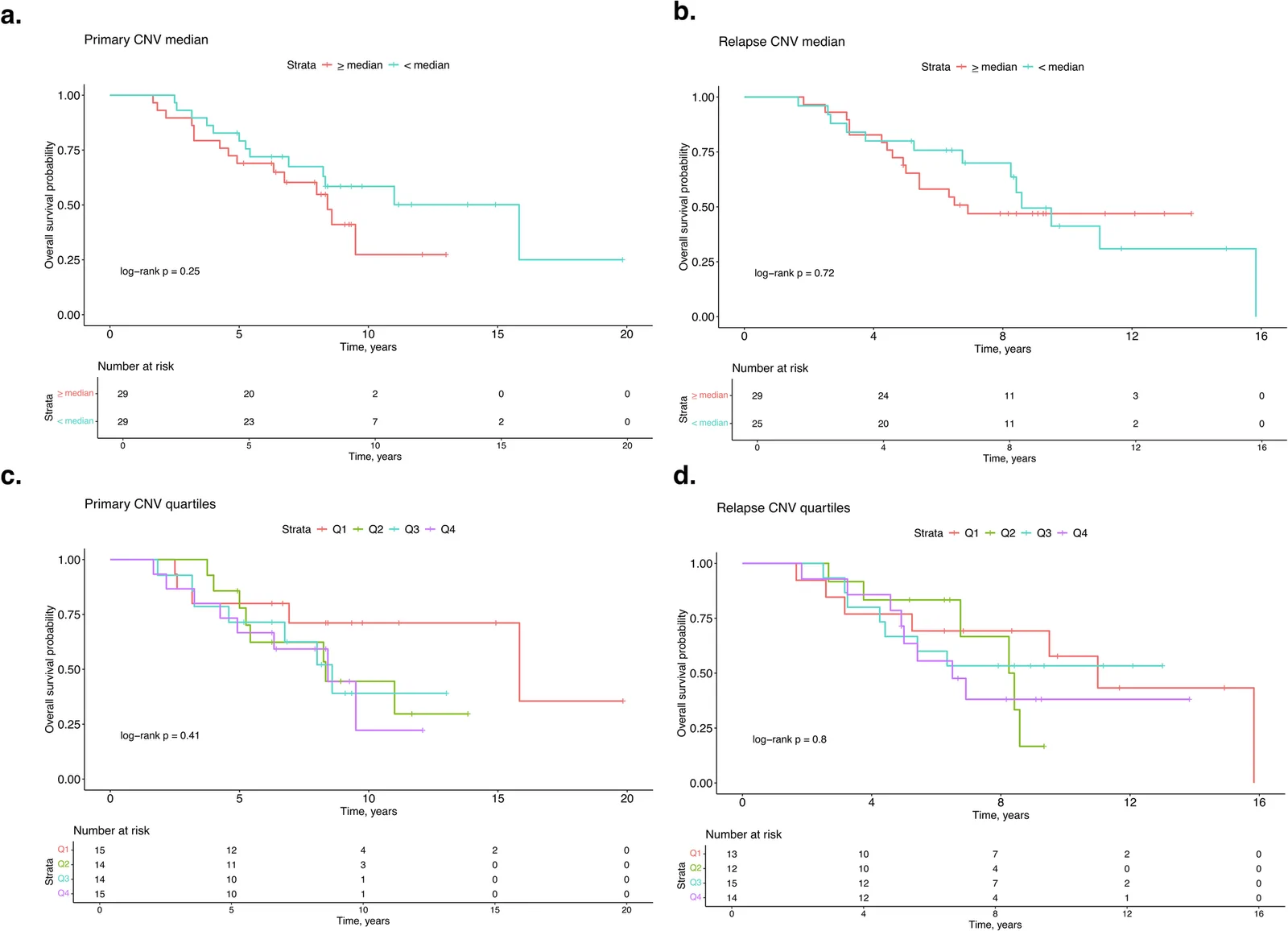
Kaplan–Meier curves demonstrating the association between the number of copy number variations and overall survival. Copy number variation (CNV) burden divided into high versus low after the median CNV in the primary (**a**) and relapse (**b**) tumors. CNV burden divided into quartiles (Q1–Q4) in the primary (**c**) and relapse (**d**) tumors.

No associations with OS were observed in the comparison of the number of CNVs in the primary (log-rank *p* = 0.25), nor relapse tumors (log-rank *p* = 0.72) of the patients, when dividing on the median cut-off (Fig. 4a and b), and neither when dividing upon quartiles for primary (log-rank *p* = 0.41), nor relapse tumors (log-rank *p* = 0.8) (Fig. 4c and d).

### TILs evaluation

Investigating the association of TMB with TILs as continuous values by Spearman correlation demonstrated no significant associations in either patients’ primary (*p* = 0.29) nor relapse tumors (*p* = 0.86). When assessing TMB at the exploratory cut-off of 10, associations were seen in the primary tumors of the patients (*p* = 0.023), with lower TILs in the group with higher TMB, but not in their relapse tumors (*p* = 0.17).

### Recurrent tumor location

**Fig. 5:**
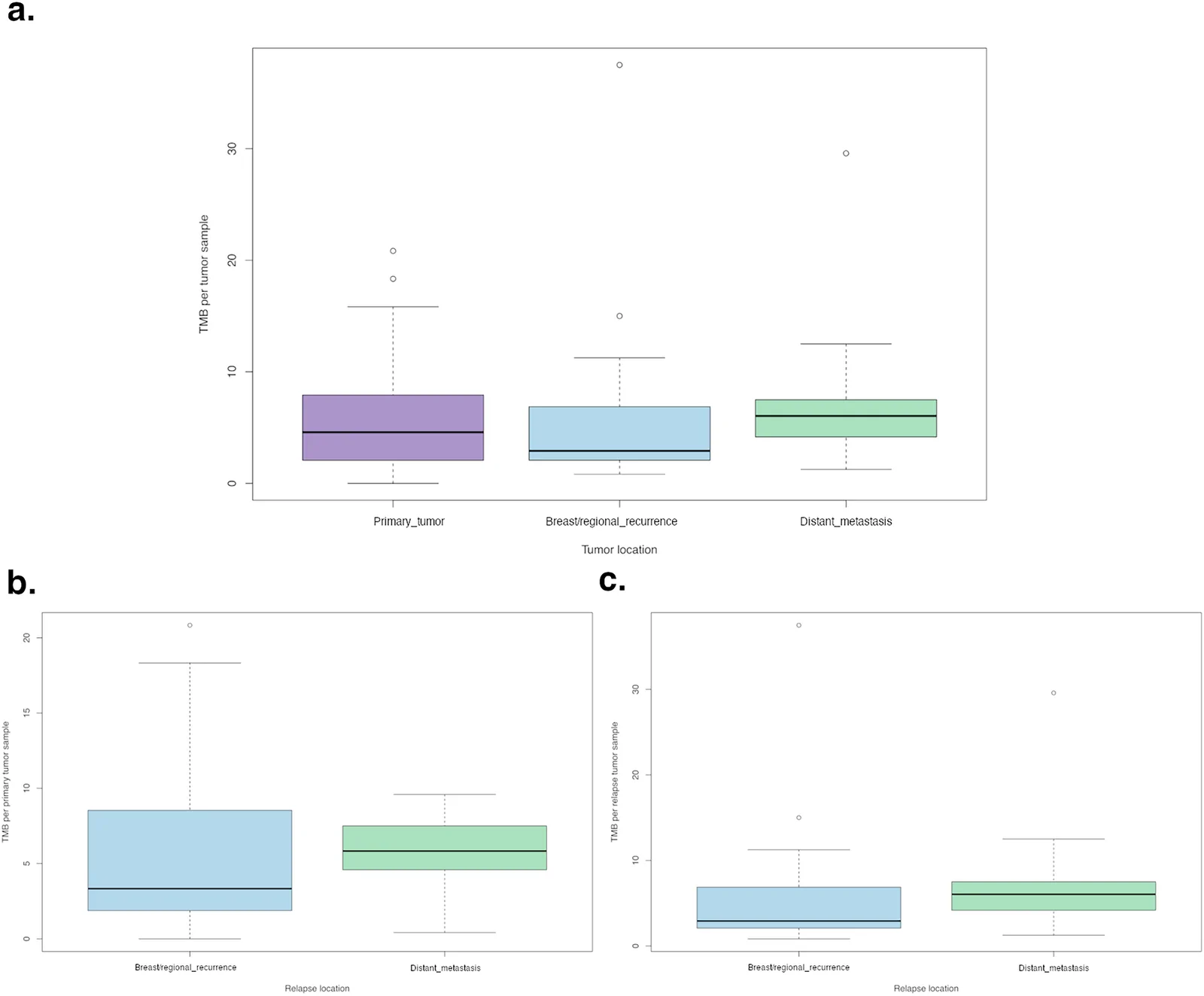
Comparisons of tumor mutational burden in patients’ tumor samples, divided according to the location of the relapse tumors. Boxplots of the tumor mutational burden (TMB) in the patients’ primary tumors, breast/regional recurrences and distant metastases (**a**). The TMB of primary tumors across the relapse location (**b**). The TMB of the relapse tumors across the relapse location (**c**).

Evaluating the possible genomic differences between breast/regional relapses or distant metastases in the patients, no significant difference was observed when comparing the TMB to the location of the primary and relapse tumors (*p* = 0.16; Fig. 5a). This was neither seen for solely primary tumors (*p* = 0.33; Fig. 5b), but a significant difference was observed for relapse tumors (*p* = 0.040; Fig. 5c). Furthermore, investigating the individual SNVs that demonstrated a nominal difference compared to the recurrence site, four SNVs (*ARHGAP35*, *ATRX*, *ETV6*, and *POT1*) were found in primary tumors, and five SNVs (*BRCA1*, *CHD3*, *COL5A1*, *MLH1*, and *SPEN*) in relapse tumors. All except SNVs in *ATRX* demonstrated a higher prevalence in patients experiencing a distant metastasis; none remained after multiple testing correction.

The number of CNVs did not show significant differences when compared to the relapse location in all tumors (*p* = 0.57), nor for primary (*p* = 0.82) or relapse tumors (*p* = 0.61). Individual CNVs did not show associations with the relapse location, in either patients' primary or relapse tumors.

### Integrated DNA/RNA analyses

**Fig. 6:**
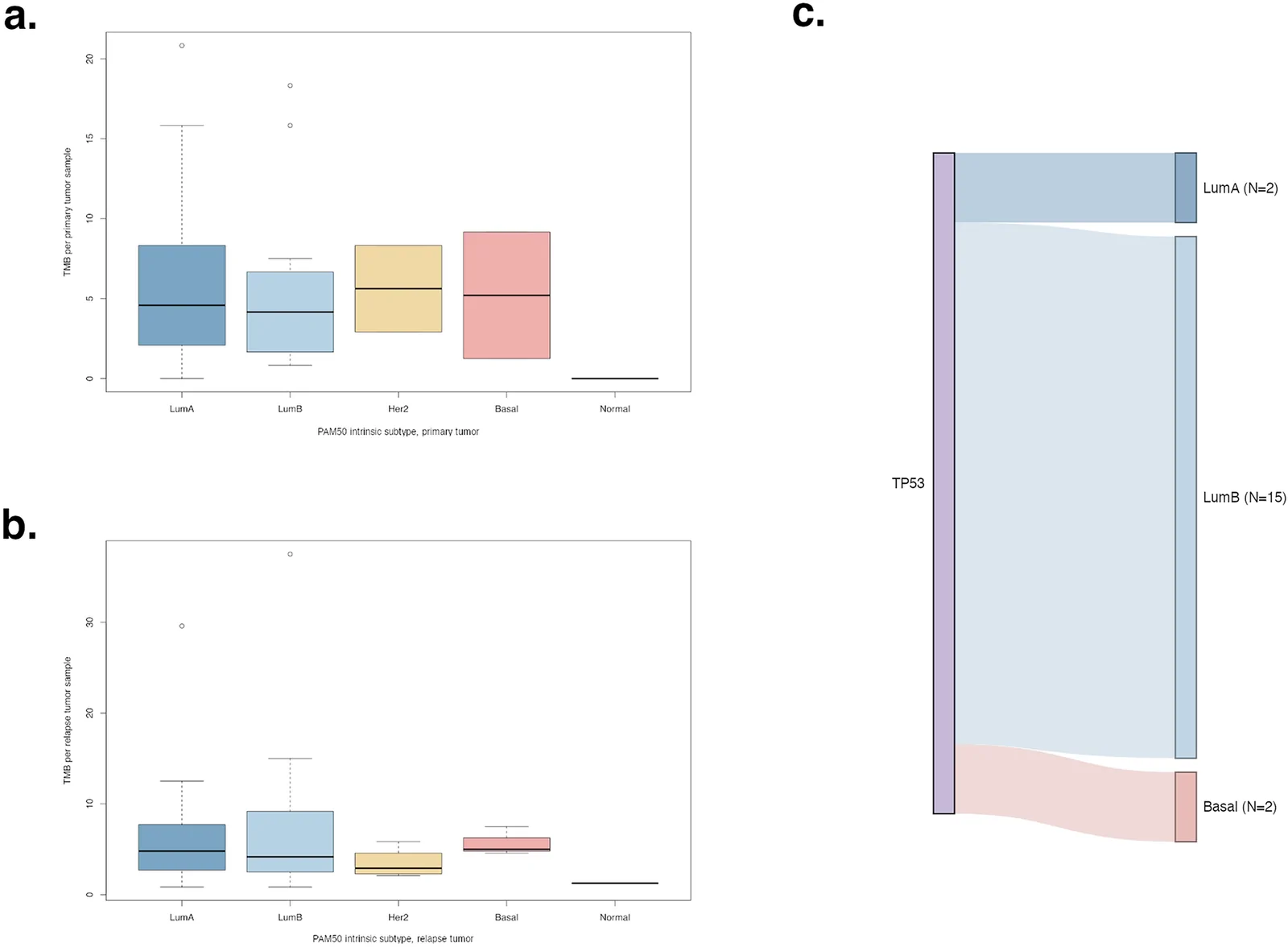
Exploration of tumor mutational burden and single-nucleotide variations in the patients’ tumor samples, and intrinsic subtypes. Boxplots of the tumor mutational burden (TMB) across intrinsic subtypes in primary tumors (**a**) and relapse tumors (**b**). Sankey diagram depicting the *TP53* single-nucleotide variations that were associated with the intrinsic subtypes in patients’ primary tumors (**c**).

Examining the transcriptomic and genomic features of the patients’ tumors, several integrated analyses were carried out. The investigation of TMB compared to PAM50 subtype analyses did not reveal significant differences between the intrinsic subtypes. Neither when analyzing the patients' primary (*p* = 0.47) nor their relapse tumors (*p* = 0.49) (Fig. 6a and b). SNVs in *TP53* in patients’ primary tumors revealed associations with the PAM50 intrinsic subtype that remained after multiple testing correction (*q* = 0.000085), demonstrating the highest prevalence (*N* = 15) in the Luminal B subtype (Fig. 6c). Out of the patients’ primary tumors in the Luminal B subtype, 13 harbored a missense variant and two harbored an in-frame deletion in *TP53*.

**Fig. 7:**
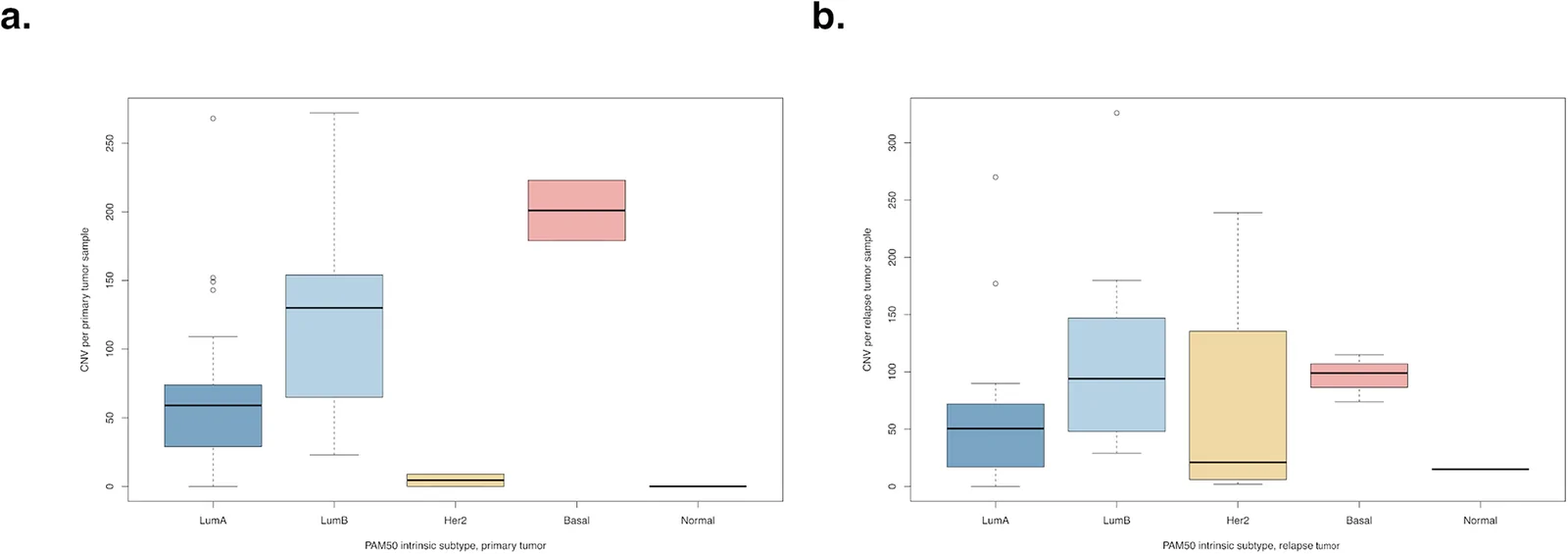
Exploration of copy number variations in the patients’ tumor samples and intrinsic subtypes. Boxplots of the copy number variations (CNVs) across intrinsic subtypes in primary tumors (**a**) and relapse tumors (**b**).

Copy numbers per sample revealed significant differences when compared to the intrinsic subtypes of both the primary (*p* = 0.00030) and relapse tumors (*p* = 0.020) of the patients (Fig. 7). The highest median number of CNVs was found in the basal subtype in both tumor groups of the patients.

Furthermore, multiple CNVs in individual genes demonstrated nominal differences across the intrinsic subtype distribution in both the primary and relapse settings; none remained after multiple testing correction.

No significant associations were seen between the TMB, the number of CNVs, or any individual SNVs or CNVs in either primary or relapse tumors with the change in subtype across the samples.

Evaluating several transcriptomic signatures with the TMB of the tumor samples, no immune signatures demonstrated significant associations. Three Hallmark gene sets: angiogenesis, coagulation, and myogenesis revealed nominal associations (*p* < 0.05) with the TMB in patients’ primary tumors, but none at BH-adjusted *q*-value < 0.1.

Moreover, 61 genes showed a correlation between the CNVs and gene expression at nominal *p*-value < 0.05, and 22 at BH-adjusted *q*-value < 0.1 (Supplementary Table 4).

## Discussion

There is currently no standardized diagnostic assessment to predict which breast cancer patients develop endocrine resistance. Transcriptomic and genomic advances over recent years have improved the diagnostic procedure through gene expression assays and genomic tests, subgrouping patients’ tumors for tailoring therapies, and thereby improving their overall outcome. However, the endocrine resistance mechanism is not yet fully understood, and resistance to novel therapies is evident, calling for further research in order to diminish the extent of patients not responding to their treatment. Our study presents the genomic and integrated transcriptomic tumor profiles of a unique cohort of endocrine-resistant patients, prior to and during ongoing endocrine therapy. The findings demonstrate potential alterations with already existing targeted treatment, highlighting means of diagnosing these.

The SNV analysis revealed mutational patterns similar to previous research, with frequent somatic mutations in *PIK3CA* and *TP53*. *TP53* was furthermore associated with the Luminal B subtype in primary tumors.

The low abundance of *ESR1* mutations and the similar *TP53* mutational frequencies between the patients’ primary and relapse tumors impeded the distinction between intrinsic and acquired resistance, which has previously been suggested^13,25,47^. Furthermore, this may suggest that these SNV frequencies do not favor clonal selection within five years of endocrine treatment. Similarly, the mutual exclusivity evaluation did not enhance any further findings. Moreover, SNVs, such as in *FAT1*, *NOTCH2*, and *BRCA* alterations, were evident. Examining the differences between the primary and relapse tumors of the patients, *BRCA1* SNVs were prevalent in 18.52% of the relapse tumors, and *BRCA2* in 5.56%. In patients’ primary tumors, the prevalence was 10.34% for each of the mutations, respectively. The survival analysis demonstrated nominal associations with a worse OS for patients with *BRCA1* SNVs in the relapse setting and *BRCA1* CNV deletions in the primary setting; however, these associations did not last when multiple testing was adjusted for. Germline BRCA mutations are well-known in breast cancer, and ~ 23% of patients with triple-negative and 5% of patients with ER-positive disease have been shown to harbor these^48^. However, less is certain about the somatic variants, though studies have shown prevalences of around 3% of *BRCA1* and *BRCA2*, respectively^49,50^. Furthermore, enriched *BRCA2* SNVs in metastatic breast cancer as compared to primary lesions have been demonstrated^18,26^. Research has additionally indicated that one-third of BRCA alterations are somatic^51^, and clinical trials are examining the effect of administering Poly(ADP-ribose) polymerase (PARP) inhibitors to patients with somatic BRCA mutations^52,53^. Our results thus demonstrate a high prevalence of BRCA alterations in this cohort of endocrine-resistant patients, particularly in the relapse setting, which could imply a greater need to further investigate their role in the resistance mechanism and potential treatment indications.

TMB was subsequently investigated, which showed differences regarding the location of relapse tumors of the patients, with higher TMB levels in distant metastases as compared to breast/regional recurrences. However, this difference was not seen in the primary setting. Previous research has shown that breast cancers have an intermediate level of TMB, where around 5% (2.9% in primary tumors and 8.4% in distant metastases) have a TMB ≥ 10 mutations/megabase (Mb)^54,55^. However, the cut-off for high and low TMB, how the tests are carried out, and how the evaluation would be utilized in the clinical setting are yet to be clarified^32,33^. A higher TMB (36% higher, paired Wilcoxon, *p* = 0.0008) has been previously reported in metastatic tumors as compared to matched primary tumors^56^, as well as a doubling of TMB for breast cancer metastases^27^. In our results, the TMB demonstrated no clear differences in the comparisons involving primary tumors, but significant differences were seen in relapse tumors. Furthermore, the Cox proportional hazard analysis revealed significant differences in the patients’ relapse tumors when comparing the continuous TMB with OS. These results thus emphasize the importance of investigating the recurrent tumor for potential clinical decisions, and further evaluation of the testing measures is needed. Moreover, when comparing the TMB to the TILs scoring, no association was seen at continuous values but could be demonstrated at the exploratory cut-off of a TMB level of ≥ 10 mutations/Mb, with higher TILs scores in the group with lower TMB levels. However, this dichotomous result remains exploratory and threshold-dependent. No transcriptomic immune-signature pattern was evident after correction, while a few non-immune Hallmark signatures showed nominal associations with the continuous TMB.

Focusing on the CNVs, the analysis revealed frequent alterations in the cohort, of which the majority were amplifications. Various amplifications were seen in genes that are well-known in cancer research, such as *AKT3*, *MYC*, *NTRK1*, and *PARP1*, and deletions in *ATM*, *CDH1*, *CHD3*, *CHEK1*, *KMT2A*, *MAP2K4*, *RB1*, and *TP53*. The average TCN per gene also demonstrated higher numbers in total, but lower levels were observed in tumor suppressors *TP53* and *RB1*, as well as in *PGR*. Evaluations of the cohort in the previous publication have demonstrated significantly lower progesterone receptor (PR) expression in the primary tumors of the resistant patients as compared to primary tumors of patients sensitive to endocrine treatment. Lower expression of PR was, as well, revealed in the relapse tumors compared to primary tumors of the resistant patients^57^. Lower PR is associated with a worse patient outcome^58^, and the results of the cohort thus show consistently low scores in protein, transcriptomic, and genomic assessments. Comparing the TCN between the patients' primary and relapse tumors, 41 genes demonstrated nominal differences, with the majority (37/41) showing higher values in the relapse setting. Some of these genes are present on the ESCAT list, such as *TP53* (ESCAT IVA), *MAP2K4* (ESCAT IVA), *CDH1* (ESCAT IVA), *ESR1* (ESCAT IIA), and, for *NCOR1* and *CHEK1*, studies are investigating their potential as inhibitors in breast cancer^30,31,59,60^. After multiple testing corrections, five genes (*CHD3*, *GPS2*, *MAP2K4*, *NCOR1*, and *TP53*) remained at BH-adjusted *q*-value < 0.1, all with higher TCN in patients’ relapse tumors. The CNVs could potentially be utilized as markers for possible treatments; however, extended assessments of larger populations are needed. Although amplifications in clinically relevant genes such as *PIK3CA* and *CDK6* showed nominal univariable associations with a worse OS, these associations were attenuated after multiple-testing correction. These observations should therefore be considered hypothesis-generating and require validation in larger cohorts before prognostic or predictive interpretations. A previous study has suggested that the genetic drivers of CNVs are established in the primary tumor, and that these alterations also remain in the metastatic lesion^12^, which could not be shown in our cohort. Moreover, research has revealed that the total number of copy number alterations has not been associated with either prognosis or metastatic site^27^. The number of CNVs in this study demonstrated a significant association with the intrinsic subtype, where differential patterns across the subtypes have previously described^61^. Furthermore, correlations between CNV and gene expression supported selected genomic findings.

Studies on paired material from patients’ tumor progression are needed to evaluate treatment response and the evolution of resistance. This study aimed to uncover the genomic patterns of a selected cohort of endocrine-resistant patients, with stringent inclusion criteria to diminish possible confounders. The inclusion criteria included patients with paired ER-positive primary and relapse tumors, HER2-negative primary tumors and no HER2-targeted treatment, and with relapse tumors during ongoing endocrine therapy. However, the selection may reduce the actual size of endocrine-resistant patients during the time period in Stockholm, and the target population may be skewed. The results thus need to be assessed in conjunction with those from larger studies and verified in a greater number of patients. The cohort criteria limit the generalizability of the results, which may not be extended to patients with relapses later than five years after primary diagnosis, patients who have ended endocrine treatment, and patients who have received newer treatment combinations, for example, CDK4/6 and PI3K inhibitors. In the univariate and univariable analyses, limitations in the statistical analyses arise, such as the risk for false positives, together with limited power for interaction analyses, subgroup analyses, and a lack of independent validation. Multiple gene-level tests were primarily examined at exploratory nominal *p*-values, and the results thus need to be interpreted with caution and further studies done, suggestively with larger data sets with adjustment for covariates. Further, limitations may arise due to the quality of the data and tumor purity. This could also interfere with the interpretations of VAF shifts, as well as shared, lost, and gained alterations. Approaches with longitudinal and current sampling, as well as single-cell profiling, may benefit further studies. Several investigations have assessed formalin-fixed paraffin-embedded (FFPE) material, concluding assessment caution but an overall reliability of results^62,63^. The panel used in the study has been proven as a robust method, generating comprehensible results^64^. The transcriptomic analyses, together with the results of the genomic alterations, demonstrate correlations between CNVs and gene expression in several genes, indicating the reliability of the results. Compliance with therapy may also affect the interpretation of the results, which was aimed to be reduced by the assessment of medical records. For the relapse tumors of the patients, several clinicopathological variables were missing and may be non-random due to the retrospective recurrent sample annotation in the medical records.

In conclusion, our study demonstrates the mutational landscape of frequent SNVs and CNVs in both the primary and paired relapse tumors of endocrine-resistant patients. Possibly, these genomic signatures may act as hypothesis-generating candidates for intervention and could perhaps serve as potential targets for already existing inhibitor treatments. In the patients’ relapse tumors, ESCAT-listed genes demonstrated a higher TCN than in the primary tumors. Further, the presence of several SNVs and CNVs showed nominal exploratory associations with OS, with a subset retained after BH correction, requiring further validation. The TMB was associated with the relapse location, and the number of CNVs and SNVs in *TP53* revealed correlations across the intrinsic subtypes. Moreover, these results may aid in further deciphering the endocrine resistance mechanism.

## Methods

### Study cohort, RNA microarray, and DNA panel sequencing

This study investigated patients with ER-positive and HER2-negative primary breast cancer, from the previously described Endoresist cohort, further detailed in previous publications^57,65^. In short, patients who developed an ER-positive relapse within five years while receiving ongoing endocrine therapy after ER-positive/HER2-negative primary breast cancer were selected as a clinically defined endocrine-resistant cohort (*N* = 62). The distinction between intrinsic and acquired endocrine resistance was not part of patient selection and was instead explored descriptively at the genomic level. DNA was extracted from archived FFPE tumor blocks of the patient's primary (*N* = 58) and relapse tumors (*N* = 54) and analyzed by a 370-gene panel at the core facility Clinical Genomics in Stockholm, Sweden (https://www.scilifelab.se/units/clinical-genomics-stockholm)^64^.

Previous assessments of the *PIK3CA* gene in these verified endocrine-resistant patients have been reported, and the cohort and methodology are further described in Schagerholm et al. ^65^ RNA was additionally extracted from archived tumor blocks of the resistant patients' primary and relapse tumors, followed by gene expression analysis. The detailed methodology and findings of the gene expression analysis of the cohort have been presented^57^. For this study, the entire 370-gene panel was assessed, including analysis of SNVs, CNVs, and correlations with gene expression investigations. Only the patients for whom data could be retrieved from the sequencing were included in the analysis. Consequently, the number of patients and samples differs from that in the previous study on the transcriptomic profiles^57^.

The final cohort thus resulted in gene expression data and sequencing data from primary tumors (*N* = 58) and relapse tumors (*N* = 54) of 62 patients. The pre-processing of the panel sequencing was carried out using the same analysis pipeline as previously described^65^, with further filtration for solely canonical mutations and those with a high or moderate impact. Thus, one of the *PIK3CA* mutations in the previous paper was not included in this study.

### Intrinsic molecular subtyping

Research-based intrinsic molecular subtypes were estimated using the subgroup-specific gene-centering method^66^, which effectively addresses the selective characteristics of ER-positive cohorts. Consequently, the correlation coefficient to the PAM50 centroids (Luminal A, Luminal B, HER2-enriched, Basal-like, and Normal-like) was calculated for each sample after gene-centering with the ER-positive subgroup percentile. All calculations were performed within the R computing environment version 4.5.0^67^, using the BreastSubtypeR R/Bioconductor package version 1.0.0^68^.

### Data analysis

In addition to routine biomarker scoring available from the point of diagnosis, tumor-infiltrating lymphocytes (TILs) were analyzed from the hematoxylin and eosin (HE)-stained tumor slides of both primary and relapse tumors by board-certified pathologists according to international guidelines from Denkert et al. ^69^ and Salgado et al. ^70^. For the comparisons in this study, positive PR status was determined by a cut-off of ≥ 10%. All patients were diagnosed as HER2-negative according to the guidelines at the point of diagnosis, and no patient received anti-HER2 treatment. For this study, HER2 status was divided into HER2-null for all patients with HER2 IHC score 0, HER2-low for HER2 IHC scores ≥ 1 without gene amplification by HER2 in situ hybridization (ISH), and HER2-amplified if gene amplified by HER2 ISH analysis^71^. Furthermore, recurrent tumor location was classified as breast/regional recurrence (ipsilateral breast or axilla, or contralateral breast) or distant metastasis.

The shift in VAFs was estimated by evaluating genes with SNVs present in at least three of the patients and shared in both the patients’ primary and relapse tumors. For multiple SNVs in the same gene in the same patient, the one with the highest mean VAF across the patients’ primary and relapse tumors was selected. The VAF shifts were tested by the Wilcoxon signed rank test, both at nominal *p*-value and Benjamini–Hochberg (BH)-adjusted *q*-value. The tumor mutational burden was calculated by dividing the number of somatic SNVs per tumor sample, as filtered defined above, by the size of the panel (2.4 Mb). TMB was exploratory evaluated as a continuous variable, per 1 mutation/Mb, and analyzed by Cox proportional hazard univariate and unadjusted model in R using the *survival* package. A threshold of 10 mutations/Mb was further tested to define high TMB, in line with previous breast cancer studies using similar panel sizes^54–56^. However, this cut-off has not been specifically validated for this panel and is used here primarily for stratification. TMB and TILs scoring as continuous values were tested by Spearman correlation. Moreover, TMB was evaluated by Spearman correlation with transcriptomic signature scores derived from published gene sets and signatures, including the Molecular Signatures Database (MSigDB) Hallmark gene sets, immune/stromal-related signatures, and immune-related gene expression signature (iGES) scores^72–75^. Gene set variation scores were estimated using gene set variation analysis (GSVA) where applicable^76^. *p*-values were adjusted for multiple testing using the BH method.

Mutual exclusivity analysis was evaluated using the Discrete Independence Statistic Controlling for Observations with Varying Event Rates (DISCOVER) test^77^. Copy number analysis was carried out on all samples using the Fraction and Allele-Specific Copy Number Estimates from Tumor Sequencing (FACETS) (v 0.6.2) tool^78^, as detailed in the previous paper^65^. The tumor ploidy was subtracted from the CNV of each gene to get an adjusted copy number. The adjusted total copy number means were evaluated at the individual CNVs and filtered at values > 1 for amplifications and < −1 for deletions. The average CNVs were assessed at a cut-off of 0.85 for tumor oncogene amplifications and −0.5 for tumor suppressor deletions. CNV burden was estimated as the number of amplifications and deletions per sample at a cut-off of absolute adjusted total copy number (TCN) of 1^79,80^. The number of CNVs was examined at both the median and the quartile levels. Moreover, gene expression of all samples was tested by linear regression against the CNVs of all samples, with multiple testing BH corrections. The paired Wilcoxon signed-rank test was used for the comparison of the TCN between the patients' primary and relapse tumors, also with BH corrections. Purity estimates from FACETS were considered when comparing the VAFs between the primary and relapse tumors.

The ESMO Scale for Clinical Actionability of molecular Targets (ESCAT) classifications for breast cancer were assessed to investigate if the results held alterations consistent with potential targets of treatment^30,31^.

In the exploratory assessments of genomic and transcriptomic results together with the clinicopathological data, continuous variables were examined using the Wilcoxon–Mann–Whitney *U* test for two independent variables and the Kruskal–Wallis test when more than two variables were involved. Fisher’s exact test was used for categorical comparisons. Survival analyses were conducted using the *survival* and *survminer* packages within the R computing environment, version 4.3.3. Overall survival (OS) was set as the endpoint and defined as the time from primary diagnosis to death or end of follow-up. OS analyses were univariate and unadjusted due to the limited sample size and intended as exploratory. Alterations present in at least five of the patients’ tumors in either the primary or relapse setting were investigated. *p*-values from the gene-level OS analyses were adjusted for multiple testing using the BH method across alteration type (SNVs or CNVs) and are reported as BH-adjusted *q*-values. Statistical tests were two-sided. Nominal *p*-values are reported and *p* < 0.05 was considered evidence of association; adjustments for multiple testing were applied with BH-adjusted *q*-values considered at *q* < 0.1. Calculations, assessments, and visualizations were carried out in Microsoft® Excel® for Microsoft 365 MSO (Version 2303, Build 16.0.16227.20202) 32-bit^81^ and in R^67^.

The study was performed in accordance with the Declaration of Helsinki. The study was approved by the Regional Ethical Review Board in Stockholm (Regionala Etikprövningsnämnden i Stockholm), Sweden. Patients provided informed consent prior to surgery for storage of tissue samples in the Stockholm Medical Biobank for clinical and research purposes. No additional informed consent was required in accordance with ethical approval in this non-interventional data collection.

## Supplementary information

Supplementary information

## Data Availability

The datasets generated and/or analyzed during the current study are not publicly available due to ethical, legal, and institutional restrictions. Data may be available from the corresponding authors on reasonable request and is subject to institutional and ethical approval, and applicable data-sharing agreements. The raw sequencing data sets analyzed in the current study are uploaded to the European Genome-phenome Archive (EGA) under accession number EGAS50000000236, accessible at https://ega-archive.org/studies/EGAS50000000236. The microarray data analyzed in the current study are uploaded to the Swedish National Data Service (SND) with the https://doi.org/10.48723/kd85-8s18. The deposited data is available through controlled access as per restrictions related to the ethical approval.

## Code availability

The analysis script and pipeline can be accessed via https://github.com/HoseinT/GMCK-cohort-analysis.
