# Supplementary material for: Genomic characterization of ER-positive primary tumors and corresponding relapses identifies potentially targetable alterations
Source: NPJ Breast Cancer. 2026 Aug 30;12:113. doi: 10.1038/s41523-026-01041-9 (PMC13526021; doi:10.1038/s41523-026-01041-9)
Supplement: Supplementary file 1 [file 41523_2026_1041_MOESM1_ESM.pdf]

Supplementary Material

Genomic characterization of ER-positive primary tumors and corresponding relapses identifies potentially targetable alterations

Caroline Schagerholm Stanev, Stephanie Robertson, Hosein Toosi, Emmanouil G. Sifakis, Jens Lagergren, Johan Hartman

Table of contents

Supplementary Table 1. Mutually exclusive genes in patients' primary tumors (a) and relapse tumors (b), filtered for $p < 0.1$.
Supplementary Table 2. Genes with differences in the total copy number (TCN) between the patients' primary and relapse tumors, as determined by the Wilcoxon signed-rank test.
Supplementary Table 3. Single-nucleotide variations (SNVs) and copy number variations (CNVs) in the tumors of the patients that were associated with a worse overall survival.
Supplementary Table 4. Genes with correlations between the normalized copy number and gene expression in the patients' tumor samples.

Supplementary Table 1. Mutually exclusive genes in patients' primary tumors (a) and relapse tumors (b), filtered for p < 0.1.

a.

**Gene_1**	**Gene_2**	**p_value**	**BH_adjusted_q_value**
FAT1	*MAP3K1*	0.0273	0.3574
GATA3	*TP53*	0.0287	0.3574
BRCA2	*PIK3CA*	0.0323	0.3574
NOTCH2	*PIK3CA*	0.0345	0.3574
CDC27	*PIK3CA*	0.0373	0.3574
FOXA1	*TP53*	0.0481	0.4349
CHD3	*FAT1*	0.0555	0.4349
ALB	*PIK3CA*	0.0606	0.5803
ATRX	*PIK3CA*	0.0606	0.5803
CDC27	*CHD4*	0.0790	0.6188
MSH6	*TP53*	0.0805	0.6188
NOTCH1	*TP53*	0.0805	0.6188
TAF1	*TP53*	0.0805	0.6188
ALK	*NOTCH2*	0.0845	0.6188
NF1	*NOTCH2*	0.0845	0.6188
KMT2D	*PIK3CA*	0.0849	0.6188
JAK1	*PIK3CA*	0.0970	0.6579

b.

**Gene_1**	**Gene_2**	**p_value**	**BH_adjusted_q_value**
CDH1	*TP53*	0.0137	0.2919
GATA3	*TP53*	0.0567	0.9386
CDC27	*SMC1A*	0.0591	0.9386
CDH1	*ZFHX3*	0.0591	0.9386
DICER1	*FAT1*	0.0603	0.9386
CDC27	*PIK3CA*	0.0685	0.9386
ALB	*ARHGAP35*	0.0766	0.9386
EP300	*PIK3CA*	0.0956	0.9386
CDH1	*NOTCH2*	0.0999	0.9386

BH=Benjamini Hochberg

**Supplementary Table 2. Genes with differences in the total copy number (TCN) between the patients' primary and relapse tumors, as determined by the Wilcoxon signed-rank test.**			
**Gene**	**p_value**	**Higher_in_tumor_sample**	**BH_adjusted_q_value**
AKT3	0.0343	Primary	0.3850
ELF3	0.0358	Primary	0.3850
FH	0.0343	Primary	0.3850
SOX9	0.0358	Primary	0.3850
ALB	0.0079	Relapse	0.2358
ANKRD11	0.0100	Relapse	0.2358
ASXL1	0.0449	Relapse	0.4057
CBL	0.0089	Relapse	0.2358
CDH1	0.0151	Relapse	0.2358
CHD3	0.0001	Relapse	0.0139
CHEK1	0.0089	Relapse	0.2358
CTCF	0.0275	Relapse	0.3377
CYLD	0.0253	Relapse	0.3227
DNMT3B	0.0449	Relapse	0.4057
ESR1	0.0218	Relapse	0.2997
FANCA	0.0249	Relapse	0.3227
GPR126	0.0024	Relapse	0.1382
GPS2	0.0001	Relapse	0.0139
HIST1H1C	0.0484	Relapse	0.4057
HIST1H1E	0.0484	Relapse	0.4057
HIST1H3B	0.0484	Relapse	0.4057
HIST1H3C	0.0484	Relapse	0.4057
HIST1H3H	0.0484	Relapse	0.4057
HLA-A	0.0484	Relapse	0.4057
INPPL1	0.0491	Relapse	0.4057
INTS4	0.0070	Relapse	0.2358
KDR	0.0131	Relapse	0.2358
KIT	0.0131	Relapse	0.2358
KMT2A	0.0151	Relapse	0.2358
LATS1	0.0070	Relapse	0.2358
MAP2K4	0.0011	Relapse	0.0787
NCOR1	0.0002	Relapse	0.0153
NQO1	0.0151	Relapse	0.2358
PARK2	0.0162	Relapse	0.2421
PDGFRA	0.0131	Relapse	0.2358
SDHD	0.0204	Relapse	0.2928
TGFBR2	0.0062	Relapse	0.2358
TP53	0.0001	Relapse	0.0139
WWOX	0.0100	Relapse	0.2358
ZBTB16	0.0151	Relapse	0.2358
ZFHX3	0.0151	Relapse	0.2358

BH=Benjamini Hochberg

**Supplementary Table 3. Single-nucleotide variations (SNVs) and copy number variations (CNVs) in the tumors of the patients that were associated with a worse overall survival.**				
**Gene**	**Sample**	**Alteration_type**	**p_value**	**BH_adjusted_q_value**
AKT3	Primary	Amplification	0.047	0.33
ALK	Primary	Amplification	0.0001	0.0047
ARHGAP35	Primary	SNV	0.027	0.37
ARID5B	Primary	Deletion	0.048	0.33
ARID5B	Primary	SNV	0.031	0.37
ATR	Primary	Amplification	0.022	0.23
B2M	Relapse	Amplification	0.0048	0.084
BCL10	Primary	Amplification	0.00013	0.005
BCL6	Primary	Amplification	0.038	0.32
BLM	Relapse	Amplification	0.041	0.33
BMPR1A	Relapse	Deletion	0.027	0.27
BRAF	Primary	Amplification	0.022	0.23
BRCA1	Primary	Deletion	0.024	0.25
BRCA1	Relapse	SNV	0.0061	0.23
BTG2	Primary	Amplification	0.028	0.27
CDC27	Primary	Deletion	0.016	0.2
CDH1	Primary	SNV	0.0055	0.23
CDK6	Primary	Amplification	0.0062	0.1
CDKN2C	Primary	Amplification	0.01	0.14
CHD3	Primary	SNV	0.0014	0.089
CSDE1	Primary	Amplification	0.0078	0.11
CTNNB1	Primary	SNV	0.037	0.37
DPYD	Primary	Amplification	0.0001	0.0047
EIF4A2	Primary	Amplification	0.038	0.32
ELF3	Primary	Amplification	0.017	0.204
EPHB1	Primary	Amplification	0.022	0.23
ERBB2	Primary	Deletion	0.0075	0.11
EZH2	Primary	Amplification	0.022	0.23
FH	Primary	Amplification	0.047	0.33
FOXL2	Primary	Amplification	0.022	0.23
FUBP1	Primary	Amplification	0.00059	0.021
GATA2	Primary	Amplification	0.015	0.199
GATA3	Primary	Amplification	0.027	0.27
GATA3	Relapse	SNV	0.046	0.37
GREM1	Relapse	Amplification	0.0048	0.084
GSK3B	Primary	Amplification	0.015	0.2
GTF2I	Primary	Amplification	0.037	0.32
H3F3A	Primary	Amplification	0.021	0.23
HGF	Primary	Amplification	0.037	0.32
HRAS	Primary	Amplification	0.042	0.33
HSD3B1	Primary	Amplification	0.0078	0.11
IDH2	Relapse	Amplification	0.041	0.33
IGF1R	Relapse	Amplification	0.0042	0.082

INPPL1	Primary	Amplification	0.0065	0.1
INTS4	Primary	Amplification	0.0028	0.07
INTS4	Relapse	Amplification	0.04	0.33
JAK1	Primary	Amplification	0.019	0.22
JUN	Primary	Amplification	0.019	0.22
KEL	Primary	Amplification	0.022	0.23
KMT2C	Primary	Amplification	0.022	0.23
KNSTRN	Relapse	Amplification	0.0048	0.084
MAP2K1	Relapse	Amplification	0.0048	0.084
MAP3K13	Primary	Amplification	0.038	0.32
MCL1	Primary	Amplification	0.047	0.33
MDM4	Primary	Amplification	0.028	0.27
MEN1	Primary	Amplification	0.0065	0.1
MGA	Relapse	Amplification	0.0048	0.084
MGA	Primary	SNV	0.038	0.37
MUTYH	Primary	Amplification	0.01	0.14
MYCL	Primary	Amplification	0.0055	0.092
NF1	Primary	Deletion	0.0052	0.088
NOTCH2	Primary	Amplification	0.016	0.2
NRAS	Primary	Amplification	0.0078	0.11
NTRK1	Primary	Amplification	0.017	0.2
PARP1	Primary	Amplification	0.021	0.23
PIK3CA	Primary	Amplification	0.016	0.2
PIK3CB	Primary	Amplification	0.022	0.23
PIK3CG	Primary	Amplification	0.012	0.17
PMS2	Primary	Amplification	0.039	0.32
POLQ	Primary	Amplification	0.015	0.2
POT1	Primary	SNV	0.029	0.37
PRKCI	Primary	Amplification	0.016	0.2
PRSS1	Primary	Amplification	0.022	0.23
PTEN	Primary	Deletion	0.025	0.25
PTPRD	Primary	Deletion	0.026	0.26
RAC1	Primary	Amplification	0.039	0.32
RAD51	Relapse	Amplification	0.0048	0.084
RHEB	Primary	Amplification	0.022	0.23
RIT1	Primary	Amplification	0.017	0.2
RPL5	Primary	Amplification	0.0001	0.0047
RPS6KA4	Primary	Amplification	0.0065	0.1
SDHAF2	Primary	Amplification	0.0065	0.1
SDHC	Primary	Amplification	0.047	0.33
SMAD2	Relapse	Deletion	0.043	0.33
SMAD3	Relapse	Amplification	0.034	0.3
SMAD4	Primary	Amplification	0.019	0.22
SMAD4	Relapse	Deletion	0.043	0.33
SMC3	Primary	Deletion	0.0014	0.045
SOX2	Primary	Amplification	0.016	0.2
SPEN	Relapse	SNV	0.0092	0.28
SPRED1	Relapse	Amplification	0.0048	0.084

SPTA1	Primary	Amplification	0.047	0.33
STAT3	Primary	Deletion	0.024	0.25
SUZ12	Primary	Deletion	0.021	0.23
TBL1XR1	Primary	Amplification	0.016	0.2
TCF12	Relapse	Amplification	0.0048	0.084
TCF7L2	Primary	Deletion	0.0014	0.045
TP63	Primary	Amplification	0.038	0.32
TPMT	Primary	Amplification	0.024	0.25

BH=Benjamini Hochberg, SNV=single-nucleotide variation

Supplementary Table 4. Genes with correlations between the normalized copy number and gene expression in the patients' tumor samples.

Gene	p_value	BH_adjusted_q_value
ARHGAP35	0.0472	0.2610
ARID5B	0.0143	0.1311
ASXL2	0.0062	0.0944
ATM	0.0264	0.1998
BAP1	0.0000	0.0019
BCL2L11	0.0007	0.0234
BCL6	0.0125	0.1311
BRD4	0.0024	0.0495
CCND1	0.0000	0.0000
CCND3	0.0426	0.2592
CDH1	0.0016	0.0354
CDKN1A	0.0192	0.1652
CDKN2A	0.0014	0.0354
CHD3	0.0388	0.2592
CHEK1	0.0001	0.0025
CIC	0.0143	0.1311
CTLA4	0.0127	0.1311
CYLD	0.0472	0.2610
DAXX	0.0040	0.0665
ELF3	0.0043	0.0685
ERBB2	0.0268	0.1998
ETV6	0.0378	0.2592
EZH2	0.0039	0.0665
FAT1	0.0014	0.0354
FGFR1	0.0000	0.0000
FGFR2	0.0003	0.0097
FLT3	0.0230	0.1832
GNA11	0.0143	0.1311
GNAS	0.0000	0.0004
GPS2	0.0414	0.2592
H3F3C	0.0383	0.2592
IDH2	0.0000	0.0019
INTS4	0.0000	0.0000
IRS2	0.0111	0.1278

KEAP1	0.0446	0.2610
KEL	0.0280	0.2038
KLF4	0.0114	0.1278
KMT2B	0.0253	0.1974
LATS1	0.0000	0.0004
LZTR1	0.0157	0.1385
MDM2	0.0110	0.1278
MED12	0.0453	0.2610
MEN1	0.0211	0.1723
MSH3	0.0475	0.2610
PDGFRA	0.0016	0.0354
PIK3CD	0.0424	0.2592
PIK3CG	0.0289	0.2057
PIM1	0.0136	0.1311
PPM1D	0.0410	0.2592
PPP4R2	0.0105	0.1278
RAD50	0.0436	0.2610
RAD51C	0.0027	0.0531
RNF43	0.0145	0.1311
ROS1	0.0417	0.2592
RPTOR	0.0008	0.0245
SDHB	0.0205	0.1716
SOCS1	0.0079	0.1100
SOX9	0.0104	0.1278
STK11	0.0072	0.1049
TAF1	0.0029	0.0534
ZBTB16	0.0084	0.1120

BH=Benjamini Hochberg
